# First-generation migrants’ use of psychotropic medication in Northern Ireland: a record linkage study

**DOI:** 10.1186/s13033-019-0334-3

**Published:** 2019-12-28

**Authors:** Tania Bosqui, Dermot O’Reilly, Ari Väänänen, Kishan Patel, Michael Donnelly, David Wright, Ciara Close, Anne Kouvonen

**Affiliations:** 1 Department of Psychology, American University of Beirut, Beirut, Lebanon; 2 Administrative Data Research Centre Northern Ireland, Centre for Public Health, Queen’s University Belfast, Belfast, UK; 3 UKCRC Centre of Excellence for Public Health (Northern Ireland), Queen’s University Belfast, Belfast, UK; 4 Finnish Institute of Occupational Health, Helsinki, Finland; 5 School of Social Policy, Sociology and Social Research, University of Kent, Canterbury, UK; 6 Faculty of Social Sciences, University of Helsinki, Helsinki, Finland; 7 SWPS University of Social Sciences and Humanities in Wroclaw, Wroclaw, Poland

**Keywords:** Mental health, Migrants, Northern Ireland, Psychotropic prescription, Access

## Abstract

**Purpose:**

There is a recent and growing migrant population in Northern Ireland. However, rigorous research is absent regarding access to mental health care by different migrant groups. In order to address this knowledge gap, this study aimed to identify the relative use of psychotropic medication between the largest first generation migrant groups in Northern Ireland and the majority population.

**Methods:**

Census (2011) data was linked to psychotropic prescriptions for the entire enumerated population of Northern Ireland using data linkage methodology through the Administrative Data Research Centre Northern Ireland (ADRC-NI).

**Results:**

Lower prescription dispensation for all psychotropic medication types, particularly antidepressants (OR = 0.35, CI 95% 0.33–0.36) and anxiolytics (OR = 0.42, CI 95% 0.40–0.44), was observed for all migrant groups with the exception of migrants from Germany.

**Conclusions:**

It is likely that the results reflect poorer access to services and indicate a need to improve access and the match between resources, services and the health and social care needs of migrants. Further research is required to identify barriers to accessing primary care and mental health services.

## Background

In multicultural societies across Europe, research has highlighted substantial and pervasive inequalities in mental health and wellbeing amongst first generation migrant groups compared to the settled majority [1, 2] though there are mixed findings in relation to country of origin, receiving society and the migration context. Compared to the settled majority, worse mental health outcomes have been found for migrants from the Caribbean [3, 4], Ethiopia [5], Surinam [6], Morocco [7], South Asia [8] and the former Soviet Union [5]; and comparable outcomes for migrants from northern to southern Europe, East Asia [3], North America, Australia, South America [5] and Turkey [7]. This pattern of results may be due to some migrant populations experiencing disproportionate levels of discrimination, isolation and social disadvantage—major risk factors for mental ill-health [9]. Migrants to low income countries [10] and asylum seekers [11] have also experienced worse outcomes. These health and well-being outcomes for migrants from low income countries tend to be associated with poorer living conditions, unemployment, limited access to schools and health care, and discrimination. The uncertainty and stress of the asylum seeking process, an increased exposure to traumatic events prior, during, and post-migration, and frequent experiences of discrimination for people who seek asylum in high income countries contributes to poor outcomes for this vulnerable group [12]. Other contextual factors, such as downward social mobility after migration, poor working conditions and unemployment [13–15], and living in an urban high deprivation neighbourhood [16], have been linked to poorer migrant mental health.

Despite the higher risks and multitude of risk factors, migrants face greater challenges in accessing mental health care than the settled majority population. These challenges include low proficiency of the majority language, fear and shame associated with disclosing mental health difficulties, lack of knowledge about how to access services, and greater distances and higher associated costs to travel to services [17]. These challenges are compounded by widespread institutional racism and discrimination in services and low cultural competence of health care staff [18]. Register-based studies found an overall lower uptake of mental health treatment by migrant groups. In Sweden, psychotropic prescription dispensations were lower for refugees than Swedish-born residents, with a comparable level reached after 10 years of residence [19]; and in Denmark, antidepressant uptake after hospitalisation for depression was found to be lower for migrant groups compared to Danish-born residents [20]. In tertiary care, however, migrants were found to have a higher risk of hospital admission compared to the Swedish born majority [21]. These contrasting findings have been explained by a greater use of emergency and involuntary health care by migrants, and an underuse of primary care and voluntary services [11].

Northern Ireland, once a region of high outward migration to other parts of the world, has seen a rapid increase in international inward migration since the accession of eight Eastern European countries to the European Union (EU) in 2004; 4.4% of all Census respondents in 2011 were born outside the United Kingdom (UK) and Republic of Ireland (RoI), compared to only 1.8% in 2001 [22]. Research is sparse regarding the mental health needs of first generation migrants living in Northern Ireland despite the increasing migrant population size and the potentially serious implications for mental health policy and service provision. Additionally, the unique context of Northern Ireland, with ongoing sectarian tensions and a high level of psychiatric disorders related to the 30 year civil conflict [23] as well as dramatic increases in racially motivated hate crimes [24], makes the generalisation of findings from other European countries and regions even more difficult. The few studies conducted so far in Northern Ireland indicated a mixed picture about the mental health of migrant groups. For example, a small-scale qualitative study found high levels of depression among the Polish-born population of Northern Ireland [25], whilst a Census-based study found lower levels of self-reported mental health problems compared to the Northern Ireland-born population [26]. This finding is in direct contrast to multiple studies in other countries that founder higher levels of self-reported mental health problems [1, 2], and was explained by a higher degree of stigma that affected the reporting of mental illness by migrant groups, and by the pre-existing high level of mental ill-health in Northern Ireland. This study investigates for the first time the level of mental health care use, measured by psychotropic medication prescriptions, by migrant groups in this Northern Ireland context.

The formation of the Administrative Data Research Centre—Northern Ireland (ADRC-NI) [27] has provided opportunities to research the mental health needs of first generation migrants on a large scale, using the entire population of Northern Ireland enumerated in the 2011 Census, linked anonymously to dispensed psychotropic prescriptions. This research capacity afforded an opportunity to accurately estimate the use of psychotropic medication by the migrant population of Northern Ireland and, in turn, to inform statutory and third sector services designed to improve the population’s mental health and enhance societal equality. Previous record-linkage research in other countries and regions have yielded large sample sizes and high data accuracy, thereby addressing the limitations of other research methodologies in the field of migrant mental health [11].

This study used large-scale administrative linked datasets to determine the relative use of psychotropic medication (antidepressants, anxiolytics and hypnotics, and anti-psychotic drugs) between the largest first generation migrant groups in Northern Ireland and the UK and RoI-born majority. Based on findings from Sweden to Denmark on low migrant psychotropic use, and the documented barriers for migrants to access mental health care in Northern Ireland [28], we expect that this study will find; (a) that migrants will use significantly less prescribed psychotropic medication than the settled majority; (b) that there will be within-group differences dependent on region of migration; and (c) that differences will narrow after adjustment for individual and neighbourhood socio-economic and demographic characteristics.

## Methods

### Data Sources

Through the ADRC-NI, this population-based study linked the entire enumerated population of Northern Ireland in the 2011 Census to individual psychotropic prescriptions using data from the Enhanced Prescribing Database (EPD) held by the Business Services Organisation (BSO).

The 2011 Census of Northern Ireland holds comprehensive and robust de-identified data on demographic and socio-economic characteristics (e.g. age, sex marital status, employment status, home ownership); migration characteristics (e.g. country of birth); and neighbourhood factors (e.g. urbanicity). The latter is maintained by the Northern Ireland Neighbourhood Information Service (NINIS). The completion of the Census is mandatory for all households in Northern Ireland, accurate on the day of the Census (27 March 2011), and is subjected to rigorous quality assurances. Just under 92% of residents adequately completed the Census, an additional 4% were captured through Health Card Registers and the remaining were imputed through a coverage and assessment process, totalling a population of 1,810,900 residents [29].

Prescription data, held by BSO, has data on prescriptions dispensed from pharmacists or dispensing doctors, including the BNF (British National Formulary) code. This study obtained information of individual prescribed medications for antidepressants, hypnotics and anxiolytics, and drugs used to treat psychoses and related disorders for a time period that contains the Census date, from 01 January 2011 until 31 December 2011.

General Practitioner (GP) appointments and prescription medications are free of charge for all those entitled to register in the Health and Social Care (HSC) system of Northern Ireland, including those seeking asylum that are supported by the National Asylum Support Service (NASS). However, asylum seekers at the time of data collection who had their asylum application refused were no longer entitled to access primary health services regardless of whether they remained in the country [30].

### Population description

The population included in this study was all non-institutionalised residents of Northern Ireland enumerated in the 2011 Census (1,672,552 records) matched to BSO data (1,587,627 records). Children and older adults (< 16 and > 64) were excluded as their use of psychotropic medication may be confounded by other health factors (567,868 records removed). All non-response (missing/edited) data were excluded prior to obtaining the dataset from NISRA as part of the data protection agreement. In total, 1,019,759 records were included in the final sample.

### Variable preparation

#### Migrant status and origin

For the purposes of this study a first-generation migrant was defined as a person resident in Northern Ireland who was born outside of Northern Ireland, the rest of the UK, and RoI. Given the unique socio-political context of Northern Ireland, a sensitivity analysis was also conducted using an alternative definition of the settled majority. In this definition, the settled majority included only those born in Northern Ireland, therefore creating a separate category for those born in the rest of the UK and RoI. Due to small numbers from some countries of origin, only the largest migrant groups were included based on a single country of birth, while the other countries were combined into larger regional categories. In total, 16 categories of migrant country or region of birth were included; Poland, Lithuania, India, USA, Germany, North Africa and Middle East, Central/Eastern/Western Africa, Southern Africa, Americas/Caribbean, China and Hong Kong, Central/Eastern/South Eastern Asia and Eastern Europe (non-EU), Southern Asia, Central/Eastern Europe (CEE), Southern Europe, Northern and Western Europe, and Oceania (for a list of included countries see Additional file 1: Table S1).

#### Psychotropic prescriptions

Psychotropic prescriptions were categorised as indicated for common mental disorders; (a) antidepressants (BNF code 4.3), and (b) anxiolytics and hypnotics (BNF code 4.1); and for psychotic disorders; c) antipsychotics including all drugs used in psychoses and related disorders (BNF code 4.2). Individuals were coded as having used a psychotropic medication if they had accessed at least one prescription in 2011, the same year as the Census.

#### Individual characteristics

Individual socio-demographic characteristics relevant for mental health were derived from the Census. These include gender (male/female), age (16–24, 25–34, 35–44, 45–54, 55–64), marital status (married, never married, separated/divorced/widowed), employment status (managerial, intermediate, small employers, routine/semi-routine, never worked/long-term unemployed, students, home ownership (owners, private renters, social renters), car ownership (0, 1, 2+) and chronic physical health problems (yes/no for at least one of: breathing difficulties, mobility difficulties, or long term pain).

#### Area characteristics

Urbanicity was measured by settlement band (urban: Belfast and Derry; intermediate; rural) based on Super Output Areas (SOA) derived from the Census.

### Data linkage

Census and prescription data were linked using anonymous one-way encryption methods by the data custodians at BSO and the Northern Ireland Statistics and Research Agency (NISRA). Anonymous de-identified data was made available to the accredited research team in a secure setting located in NISRA. All output was screened by Research Support Officers in NISRA for non-identifiability before being approved for release. Ethical approval was obtained from the Office for Research Ethics Committee Northern Ireland (ORECNI; Ref: 15/WM/0212), the Research Ethics Committee for the School of Medicine, Dentistry and Biomedical Sciences at Queen’s University Belfast (Ref: 14/54), and the Administrative Data Research Network (ADRN) Approvals Panel (Ref: 2014/008); and conform to the principles embodied in the 1964 Declaration of Helsinki and its later amendments.

### Statistical analysis

Descriptive analyses of the sample included comparisons (frequencies) of socio-demographic characteristics between migrant groups and the settled majority, and the testing of any differences between groups (using χ^2^). The logistic regressions (with CIs at 95%) were run for all psychotropic medications and all migrant groups compared to the settled majority, using four models. The first model was unadjusted, the second was adjusted for demographic factors (age, gender, and marital status), the third was adjusted for physical health, and the final model was adjusted fully for socio-demographic factors (employment, car availability, housing tenure, and urbanicity). These models provided an overall analytical picture of differences in prescriptions and the impact of socio-demographic and economic factors. Next, the adjusted models were applied to different psychotropic medication types broken down by migrant group in order to observe any group differences, and prescription type differences, in the use of psychotropic medications.

A sensitivity analysis was conducted that included only people who were born in Northern Ireland in the settled majority group. No significant difference was found in the number of psychotropic prescriptions between different definitions of the settled majority (*p *= 0.10).

## Results

### Population characteristics

A total of 1,019,759 people were included, 49,342 of whom were born outside of the UK and RoI (4.8%). Table 1 shows that migrants had a higher proportion of employment in routine work and of private renting than the majority settled population. A breakdown by migrant group (see Additional file 1: Table S2), showed that migrants from Poland, Lithuanian and other Central and Eastern European (CEE) countries had the highest proportion of routine work and private renting, whilst migrants from Germany, the USA, Americas/Caribbean, and Northern and Western Europe had the highest proportion of home ownership after the settled majority.

**Table 1:** Population characteristics for all migrants compared to the settled majority

	Northern Ireland	Great Britain and Republic of Ireland	Migrants
Number of persons (%)	898,945	71,472	49,342
	88.2	7.0	4.8
Sex			
Male	48.7	46.2	47.1
Female	51.3	53.8	52.9
Age			
16–24	19.1	11.7	13.1
25–34	19.6	16.1	37.9
35–44	20.8	25.1	26.9
45–54	22.5	26.7	15.6
55–64	18.0	20.4	6.5
Marital status			
Never married	43.1	32.4	38.5
Married	45.7	53.4	51.1
Separated/divorced/widowed	11.2	14.2	10.4
NS – SEC			
Managerial	29.0	36.5	28.1
Intermediate	13.0	12.8	8.3
Small employers	9.2	7.8	5.8
Routine/semi-routine	29.5	27.4	42.2
Never worked/unemployed	9.2	8.2	7.7
Students	10.1	7.3	8.0
Car availability			
0	13.2	14.4	22.5
1	32.0	35.2	45.9
2 or more	54.8	50.4	31.6
Housing tenure			
Owns outright	75.5	68.2	35.7
Private renting	11.5	19.1	54.3
Social renting	13.0	12.7	10.1
Settlement band			
Urban	19.8	19.9	15.2
Intermediate	46.5	48.6	59.3
Rural	33.7	31.5	15.5

#### Prescription of psychotropic medication

In total, 11.8% of migrants were prescribed a psychotropic medication compared to 24% of the settled majority. A breakdown of prescription type by migrant group is displayed in Additional file 1: Table S3. In both migrant and settled populations, prescriptions were higher for females (settled population = 30.3%, migrants = 14.9%) than males (settled population = 17.4%, migrants = 8.2%). An interaction effect was tested for a modifying effect of gender on the association between region of birth and psychotropic medication use. No significant interaction effect was found (*p *= 0.08) and therefore logistic regression models were not stratified by gender.

The results showed a lower likelihood of prescriptions for migrants compared to the settled majority in Northern Ireland; with migrants almost 60% (OR = 0.42, CI 95% 0.41–0.43) less likely to have a prescription in the unadjusted model (see Table 2). The likelihood reduced further after adjustment for socio-demographic, economic, and health covariates (OR = 0.37, CI 95% 0.36–0.38). A breakdown by migrant group (see Table 3) showed that the lower likelihood is consistent across migrant groups, with the exception of a comparable likelihood for migrants from Germany (OR = 0.95, CI 95% 0.86–1.04). The lowest use of psychotropic medication was found for migrants from Central to Eastern Europe (OR = 0.19, CI 95% 0.17–0.21), Lithuania (OR = 0.20, CI 95% 0.18–0.22), and India (OR = 0.21, CI 95% 0.18–0.24).

**Table 2:** Logistic regression for dispensation of any psychotropic medication

	Model 1 OR (CI)	Model 2 OR (CI)	Model 3 OR (CI)	Model 4 OR (CI)
Migrant status (ref: settled)				
All migrants	0.42 (0.41–0.43)	0.45 (0.43–0.46)	0.47 (0.46–0.48)	0.37 (0.36–0.38)
Age (ref: 16–24)				
25–34	–	2.69 (2.63–2.74)	2.68 (2.62–2.73)	2.14 (2.09–2.19)
35–44	–	4.51 (4.42–4.61)	4.26 (4.17 – 4.35)	3.38 (3.29 – 3.46)
45– 4	–	5.83 (5.71–5.96)	5.10 (4.99 – 5.21)	4.04 (3.94 – 4.14)
55–64	–	6.68 (6.53–6.83)	5.08 (4.96 – 5.20)	3.93 (3.82 – 4.03)
Sex (ref: male)				
Female	–	2.06 (2.04–2.08)	2.12 (2.10 – 2.14)	2.11 (2.09 – 2.14)
Marital status (ref: married)				
Never married	–	1.55 (1.53–1.57)	1.46 (1.44–1.48)	1.03 (1.02 – 1.05)
Separated/divorced/widowed	–	2.33 (2.30–2.36)	2.15 (2.12–2.18)	1.47 (1.45 – 1.49)
Poor physical health (ref: no)				
Yes	–	–	2.99 (2.96–3.03)	2.62 (2.58–2.65)
NS – SEC (ref: managerial)				
Small employers	–	–	–	1.25 (1.23–1.27)
Intermediate	–	–	–	1.11 (1.10–1.14)
Routine/semi-routine	–	–	–	1.42 (1.40–1.43)
Never worked/unemployed	–	–	–	1.53 (1.50–1.56)
Students	–	–	–	0.72 (0.71–0.76)
Car availability (ref: 2 +)				
1	–	–	–	1.31 (1.29–1.32)
0	–	–	–	1.71 (1.68–1.74)
Housing tenure (ref: owns)				
Private renting	–	–	–	1.33 (1.31–1.36)
Social renting	–	–	–	1.69 (1.66–1.72)
Urban–rural living (ref: urban)				
Intermediate	–	–	–	0.97 (0.95–1.00)
Rural	–	–	–	0.89 (0.88–0.90)

Model 1: Unadjusted

Model 2: Adjusted for demographic factors

Model 3: Plus adjustment for poor physical health

Model 4: Plus adjustment for socioeconomic factors

*OR* odds ratio, *CI* confidence intervals

**Table 3:** Fully adjusted model for different psychotropic medications by migrant group

	All prescriptions OR (CI)	Anti-depressants OR (CI)	Anxiolytics and hypnotics OR (CI)	Anti-psychotics OR (CI)
Migrant status (ref: settled)				
All migrants	0.37 (0.36–0.38)	0.35 (0.33–0.36)	0.42 (0.40–0.44)	0.37 (0.34–0.41)
Poland	0.24 (0.23–0.26)	0.21 (0.20–0.23)	0.29 (0.26–0.31)	0.18 (0.14–0.24)
Lithuania	0.20 (0.18–0.22)	0.18 (0.16–0.21)	0.22 (0.19–0.26)	0.19 (0.13–0.29)
India	0.21 (0.18–0.24)	0.16 (0.13–0.19)	0.28 (0.23–0.34)	0.29 (0.18–0.49)
USA	0.79 (0.71–0.89)	0.78 (0.68–0.88)	0.66 (0.56–0.77)	0.94 (0.68–1.29)
Germany	0.95 (0.86–1.04)	0.91 (0.83–1.01)	1.00 (0.89–1.12)	0.91 (0.71–1.16)
North Africa and Middle East	0.59 (0.49–0.70)	0.55 (0.46–0.67)	0.56 (0.44–0.71)	0.47 (0.27–0.82)
C/E/W Africa	0.54 (0.47–0.62)	0.49 (0.42–0.56)	0.59 (0.50–0.70)	0.65 (0.46–0.93)
Southern Africa	0.83 (0.71–0.96)	0.73 (0.62–0.87)	0.82 (0.67–1.00)	0.89 (0.58–1.38)
The Americas/Caribbean	0.79 (0.71–0.88)	0.79 (0.70– 0.89)	0.81 (0.71–0.93)	0.72 (0.52–1.01)
China and Hong Kong	0.23 (0.20–0.26)	0.19 (0.16–0.23)	0.30 (0.24–0.36)	0.29 (0.18–0.47)
C/E/SE Asia and E. Europe	0.23 (0.20–0.26)	0.19 (0.16–0.22)	0.32 (0.27–0.37)	0.21 (0.13–0.32)
Southern Asia	0.54 (0.45–0.65)	0.45 (0.37–0.56)	0.55 (0.43–0.71)	0.89 (0.56–1.40)
C/E Europe (CEE)	0.19 (0.17–0.21)	0.17 (0.15–0.19)	0.19 (0.16–0.23)	0.16 (0.10–0.24)
Southern Europe	0.60 (0.54–0.66)	0.60 (0.54–0.68)	0.61 (0.53–0.70)	0.48 (0.35–0.66)
Northern and Western Europe	0.49 (0.42–0.57)	0.49 (0.41–0.58)	0.53 (0.43–0.66)	0.39 (0.22–0.69)
Oceania	0.75 (0.65–0.86)	0.70 (0.60–0.82)	0.79 (0.66–0.96)	0.92 (0.63–1.36)

*C* central, *CI* confidence intervals, *E* eastern, *OR* odds ratio, *SE* south eastern, *W* western

In terms of prescription type Table 3 also shows that all psychotropic types were prescribed less for migrants than the settled majority (anxiolytics OR = 0.42, CI 95% 0.40–0.44; antidepressants OR = 0.35, CI 95%0.33–0.36; and antipsychotics OR = 0.37, CI 95% 0.34–0.41). For prescription type broken down by migrant group, the same pattern was found for anxiolytics and antidepressants as for psychotropic medication in general. However, for antipsychotics there are some notable differences. Unlike for anxiolytics/hypnotics and antidepressants, migrants from the USA, Southern Africa, the Americas/Caribbean, Southern Asia, and Oceania had a comparable likelihood of having an antipsychotic prescription dispensed. Migrants from Poland, Lithuania and other Central and Eastern European countries, were over 80% less likely to be prescribed antipsychotic medication compared to the settled majority (OR = 0.18, CI 95% 0.14–0.24; OR = 0.19, CI 95% 0.13–0.29; OR = 0.16, CI 95% 0.10–0.24; respectively).

## Discussion

This is the first population-based administrative-linked data study to examine access to mental health care by analysing psychotropic prescriptions for first generation migrants resident in Northern Ireland. The results showed a consistently lower level of dispensation of psychotropic drugs for migrants compared to the settled majority, with the exception of a comparable dispensation level for migrants from Germany, and the dispensation specifically of antipsychotic drugs for a number of other groups. These findings are in keeping with studies in Sweden and Denmark [19, 20] and may indicate that similar barriers to accessing mental health treatment exist in Northern Ireland.

The lower use of psychotropic medication by most migrants groups, particularly regarding antidepressants and anxiolytics/hypnotics, is likely to reflect poorer access to services. Barriers to care may include poorer knowledge about how to access services, greater stigma in relation to help-seeking for mental distress, more negative beliefs about medication [31], and diverse cultural and religious conceptualisations of mental distress and treatment [32], as well as structural barriers to accessing services such as poor cultural competencies among service providers, discrimination and prejudice at the point of access [33], limited availability of translators [34], and overall poor service provision [31]. According to Polish migrants in Northern Ireland who were interviewed as part of a qualitative study, language barriers and stigma were the key challenges to accessing mental health services [25]. Socio-economic factors per se do not appear to contribute to explanations about lower psychotropic medication use. Adjustment for these factors did not affect the differences between migrant and non-migrant groups.

The improved accessibility for antipsychotic medication compared to antidepressants and anxiolytics for many migrant groups is likely to reflect the severity and easier ‘visibility’ of the illness that requires this treatment. However, the comparable level of psychotropic prescription dispensation, of all types, for migrants from Germany may reflect higher social and economic status and opportunity in the group, including higher home ownership than other migrant groups. It may also indicate lower stigma and greater belief in the effectiveness of medication for mental distress in this group. This interpretation is supported by the results of research about stigma [35] and negative beliefs about medication [36], both of which tend to be associated with less access and use of mental health services.

In the context of Northern Ireland, these findings are of particular importance. Migration from countries outside Great Britain and RoI is a relatively new, and growing, phenomenon for Northern Ireland. Migrants join a post-conflict society that continues to struggle with the challenges of healing hurts and divisions. Often, available housing and accommodation for migrants is located in divided and polarised community settings that are relatively impoverished. Some migrants have been the target of increasing racist hate crimes [24] and there is a degree of anti-migrant discourse that is fuelled by ongoing sectarianism and the fallout of the 2007–2008 economic crisis. Qualitative evidence indicates that many migrants do not feel they ‘belong’ in Northern Ireland [25, 28], which affects all aspects of their lives, including health and health care. The lower use of psychotropic drugs found in this study may reflect a degree of disaffection from Northern Irish civil society. These findings highlight the need for further examination of the challenges and factors that affect migrant health and wellbeing in Northern Ireland as well as avenues to address them.

### Strengths and limitations

This study uses data linkage methodology and draws on robust and comprehensive administrative data about the whole enumerated population of Northern Ireland in the 2011 Census. It is the first study to use these data to identify psychotropic prescription utilisation between migrant groups in comparison to the settled majority population. Despite its large population-based sample and high quality real-world data, the study has a number of limitations. The dataset assumes that migrant groups had an opportunity to respond to the Census, and that they filled out the Census to the same extent as the settled majority. No undocumented migrants are included in the data, and it is unclear to what extent this may affect the results as little is known about the number of undocumented migrants in Northern Ireland. However, given the already poor access to services, undocumented migrants are likely to have even poorer access to services than documented migrants. This is supported by European data on poor access to primary care services for irregular or undocumented migrants [37]. There is also some indication that, given the structural barriers to care, migrants from nearby countries in the EU may return home to access medical treatment [25] and this may contribute to lower levels of uptake in Northern Ireland. Finally, additional characteristics of time since most recent arrival in Northern Ireland, reason for migration, and proficiency in English could not be included due to low response rate, insufficient sample sizes, and no significant effect respectively. Exploration of different reasons for migration (such as asylum seeking and economic migration) was particularly difficult in the administrative data set due to small numbers in some groups. In terms of defining access to mental health care, prescriptions of psychotropic medications may reflect a range of different difficulties and are not synonymous with mental health care. Some migrant groups may be more likely to seek treatment in other service types, such as psychological therapy, family and community support, or religious healers [38]. Antidepressant medication can also be prescribed for related health conditions such as chronic pain and sleep problems [39]. This study is therefore reflective only of medical treatment of distress and not of other forms of mental health care or specific psychiatric conditions.

### Implications

The consistent findings of lower use of psychotropic medications for most migrant groups in Northern Ireland is likely to be a result of poorer access to services. Improved access to care is imperative in order to improve the wellbeing of these groups. Improving access may involve additional training for staff in providing culturally sensitive care, provision of cultural mediators and translators, recruiting a diverse health practitioner team, creating partnerships with community migrant groups, and implementing preventative interventions for at-risk groups such as refugees and asylum seekers. Improvement can also be made at a social level, such as interventions that address the disproportionate disadvantage and acculturation stress affecting first generation migrants [39]. Attention is needed at the policy level in order to ensure that these research-informed ways of improving access to services by migrants are implemented and delivered. The need to improve access to mental health care is particularly important given the growing number of migrant populations, and the toll that this may take on the health and social care system in the future, if the needs-services gap is not met. Further research is required to understand the barriers to care, in order to address this inequality in service provision.

## Conclusions

This large study linked Census data to psychotropic prescriptions for the entire enumerated population of Northern Ireland and found lower dispensation of psychotropic medication, particularly antidepressants and anxiolytics, for first generation migrants compared to the settled majority. It is likely that the results reflect poorer access to services and indicate a need to improve access and the match between resources, services and the health and social care needs of migrants.

## Supplementary information

**Additional file 1: Table S1.** Country categories. **Table S2.** Population characteristics by migrant group compared to the settled majority. **Table S3.** Prescriptions by migrant group compared to the settled majority.

## Data Availability

The datasets generated and analysed during the current study are not publicly available as they are protected by the Northern Ireland Statistics and Research Agency and can only be provided with their review and permission.

## Abbreviations

ADRC-NI: Administrative Data Research Centre Northern Ireland
BNF: British National Formulary
BSO: Business of Organisation
CEE: Central and Eastern European
CI: confidence intervals
EU: European Union
GP: general practitioner
HSC: health and social care
NASS: National Asylum Support Service
NINIS: Northern Ireland Neighbourhood Information Service
NISRA: Northern Ireland Statistics and Research Agency
OR: odds ratio
RoI: Republic of Ireland
SOA: Super Output Areas
UK: United Kingdom
