# Supplementary material for: First-generation migrants’ use of psychotropic medication in Northern Ireland: a record linkage study
Source: Int J Ment Health Syst. 2019 Dec 28;13:77. doi: 10.1186/s13033-019-0334-3 (PMC6935113; doi:10.1186/s13033-019-0334-3)
Supplement: Supplementary file 1 — Additional file 1: Table S1. Country categories. Table S2. Population characteristics by migrant group compared to the settled majority. Table S3. Prescriptions by migrant group compared to the settled majority. [file 13033_2019_334_MOESM1_ESM.docx]

Additional file 1

Table S1: Country categories

| **Region and country** | **n** |
| --- | --- |
|  |  |
| **United Kingdom and Republic of Ireland** | **1526159** |
| Ireland | 32275 |
| Island of Ireland (Not otherwise specified) | 26 |
| England | 54229 |
| Northern Ireland | 1423401 |
| Scotland | 13434 |
| Wales | 2133 |
| Great Britain (Not otherwise specified) | 41 |
| United Kingdom (Not otherwise specified) | 46 |
| Isle of Man | 337 |
| Guernsey | 102 |
| Jersey | 112 |
| Channel Islands (Not otherwise specified) | 23 |
|  |  |
| **Poland** | **14995** |
|  |  |
| **Lithuania** | **5478** |
|  |  |
| **India** | **3899** |
|  |  |
| **United States** | **3371** |
|  |  |
| **Germany** | **3066** |
|  |  |
| **Northern Africa and the Middle East** | **1440** |
| Algeria | 105 |
| Egypt | 161 |
| Libya | 64 |
| Morocco | 80 |
| Sudan | 99 |
| Tunisia | 37 |
| Bahrain | 18 |
| Iraq | 60 |
| Israel | 47 |
| Jordan | 49 |
| Kuwait | 38 |
| Lebanon | 41 |
| Oman | 12 |
| Occupied Palestinian Territories | 25 |
| Saudi Arabia | 210 |
| Syria | 26 |
| Turkey | 284 |
| United Arab Emirates | 54 |
| Yemen | 30 |
|  |  |
| **Central, Eastern and Western Africa** | **2061** |
| Ethiopia | 21 |
| Kenya | 239 |
| Malawi | 64 |
| Mauritius | 54 |
| Mozambique | 29 |
| Somalia | 71 |
| Uganda | 66 |
| Tanzania | 58 |
| Zambia | 231 |
| Zimbabwe | 529 |
| Cape Verde | 16 |
| Ghana | 100 |
| Guinea-Bissau | 92 |
| Ivory Coast | 20 |
| Nigeria | 374 |
| Sierra Leone | 18 |
| Angola | 41 |
| Cameroon | 12 |
| Congo (Democratic Republic) | 13 |
| Sao Tome and Principe | 13 |
|  |  |
| **Southern Africa** | **1463** |
| Botswana | 24 |
| South Africa | 1439 |
|  |  |
| **Americas and the Caribbean** | **2934** |
| Cuba | 13 |
| Jamaica | 84 |
| Trinidad and Tobago | 47 |
| Argentina | 57 |
| Brazil | 293 |
| Chile | 42 |
| Colombia | 44 |
| Ecuador | 24 |
| Falkland Islands | 15 |
| Guyana | 48 |
| Peru | 77 |
| Venezuela | 39 |
| Belize | 19 |
| Guatemala | 19 |
| Mexico | 72 |
| Bermuda | 86 |
| Canada | 1918 |
| North America (Not otherwise specified) | 10 |
| Other North America | 27 |
|  |  |
| **China and Hong Kong** | **2979** |
| China | 1472 |
| Hong Kong (Special administrative region of China) | 1478 |
| Macao (Special administrative region of China) | 29 |
|  |  |
| **Central, Eastern and South Eastern Asia and Eastern Europe (non-EU)** | **5163** |
| Brunei | 15 |
| Malaysia | 538 |
| Burma | 22 |
| Indonesia | 37 |
| Philippines | 2422 |
| Singapore | 233 |
| Thailand | 351 |
| East Timor | 593 |
| Vietnam | 125 |
| Kazakhstan | 33 |
| Japan | 112 |
| Korea (South) | 76 |
| Taiwan | 65 |
| Belarus | 43 |
| Moldova | 42 |
| Russia | 257 |
| Ukraine | 175 |
| Union of Soviet Socialist Republics (Not otherwise specified) | 24 |
|  |  |
| **Southern Asia** | **1159** |
| Bangladesh | 283 |
| Iran | 215 |
| Pakistan | 477 |
| Nepal | 80 |
| Sri Lanka | 104 |
|  |  |
| **Central and Eastern Europe (CEE)** | **6047** |
| Bulgaria | 448 |
| Czech Republic | 492 |
| Czechoslovakia (Not otherwise specified) | 16 |
| Hungary | 671 |
| Romania | 764 |
| Slovakia | 1852 |
| Latvia | 1693 |
| Estonia | 73 |
| Croatia | 26 |
| Slovenia | 12 |
|  |  |
| **Southern Europe** | **3267** |
| Canary Islands | 21 |
| Gibraltar | 103 |
| Greece | 117 |
| Italy | 413 |
| Kosovo | 34 |
| Malta | 178 |
| Portugal | 1611 |
| Serbia | 16 |
| Spain (Except Canary Islands) | 36 |
| Spain (Not otherwise specified) | 455 |
| Cyprus (Not otherwise specified) | 283 |
|  |  |
| **Northern and Western Europe** | **1772** |
| Austria | 89 |
| Belgium | 151 |
| France | 654 |
| Luxembourg | 12 |
| Netherlands | 425 |
| Switzerland | 88 |
| Denmark | 82 |
| Finland | 60 |
| Iceland | 17 |
| Norway | 70 |
| Sweden | 124 |
|  |  |
| **Oceania** | **1828** |
| Australia | 1365 |
| Fiji | 29 |
| New Zealand | 434 |

Table S2: Population characteristics by migrant group compared to the settled majority

|  | | Settled Majority Population | All Migrants | Migrants by Region of Birth | | | | | | | | | | | | | | | |
| --- | --- | --- | --- | --- | --- | --- | --- | --- | --- | --- | --- | --- | --- | --- | --- | --- | --- | --- | --- |
|  |  |  |  | Poland | Lithuania | India | USA | Germany | North Africa and Middle East | C/E/W Africa | Southern Africa | Americas and the Caribbean | China and Hong Kong | C/E/SE Asia and E Europe | Southern Asia | C/E Europe | Southern Europe | Northern and Western Europe | Oceania |
| **Number of persons** | | 970,417 | 49,342 | 12,246 | 4,552 | 2,907 | 2,165 | 2,668 | 1,168 | 1,738 | 1,207 | 2,285 | 2,625 | 4,243 | 973 | 5,142 | 2,603 | 1,375 | 1,445 |
|  | % | 95.3 | 4.8 | 24.8 | 9.2 | 5.9 | 4.4 | 5.4 | 2.4 | 3.5 | 2.4 | 4.6 | 5.63 | 8.6 | 2.0 | 10.4 | 5.3 | 2.8 | 2.9 |
|  |  |  |  |  |  |  |  |  |  |  |  |  |  |  |  |  |  |  |  |
| **Sex** | |  |  |  |  |  |  |  |  |  |  |  |  |  |  |  |  |  |  |
|  | Male | 48.5 | 47.1 | 48.8 | 42.0 | 51.7 | 41.0 | 42.5 | 67.8 | 49.8 | 44.9 | 40.6 | 47.6 | 42.1 | 59.2 | 46.8 | 51.9 | 40.2 | 47.1 |
|  | Female | 51.5 | 52.9 | 51.2 | 58.0 | 48.3 | 59.0 | 57.5 | 32.2 | 50.2 | 55.1 | 59.4 | 52.5 | 57.9 | 40.8 | 53.2 | 48.1 | 59.8 | 52.9 |
| **Age** | |  |  |  |  |  |  |  |  |  |  |  |  |  |  |  |  |  |  |
|  | 16 – 24 | 16.6 | 13.1 | 11.2 | 18.6 | 4.3 | 24.7 | 16.8 | 14.3 | 9.5 | 18.6 | 9.4 | 16.0 | 9.7 | 9.3 | 13.3 | 13.8 | 12.5 | 14.9 |
|  | 25 – 34 | 19.4 | 37.9 | 55.4 | 47.0 | 39.2 | 19.3 | 26.5 | 28.2 | 21.9 | 31.0 | 24.4 | 21.5 | 23.3 | 26.8 | 49.4 | 27.2 | 27.6 | 21.2 |
|  | 35 – 44 | 21.1 | 26.9 | 21.6 | 17.9 | 36.5 | 22.2 | 29.1 | 29.2 | 31.4 | 27.8 | 26.6 | 24.5 | 45.1 | 32.2 | 22.8 | 29.4 | 28.9 | 32.3 |
|  | 45 – 54 | 22.8 | 15.6 | 8.5 | 12.7 | 12.5 | 22.4 | 19.9 | 19.4 | 24.3 | 13.6 | 27.3 | 25.3 | 16.4 | 22.9 | 11.0 | 20.9 | 18.6 | 25.0 |
|  | 55 – 64 | 18.2 | 6.5 | 3.3 | 3.8 | 7.5 | 11.4 | 7.7 | 9.0 | 13.0 | 9.0 | 12.4 | 12.8 | 5.6 | 8.8 | 3.5 | 8.6 | 12.4 | 6.7 |
| **Marital Status** | |  |  |  |  |  |  |  |  |  |  |  |  |  |  |  |  |  |  |
|  | Never Married | 42.3 | 38.5 | 40.9 | 48.2 | 13.4 | 40.0 | 47.5 | 29.7 | 31.5 | 42.5 | 31.5 | 32.6 | 26.1 | 20.7 | 51.4 | 48.6 | 39.7 | 38.7 |
|  | Married | 46.3 | 51.1 | 49.8 | 35.8 | 83.6 | 50.3 | 38.8 | 58.7 | 54.7 | 47.0 | 56.5 | 60.3 | 65.9 | 72.2 | 37.1 | 37.0 | 49.8 | 52.0 |
|  | Separated/Divorced/ Widowed | 11.4 | 10.4 | 9.3 | 16.0 | 3.0 | 9.7 | 13.7 | 11.6 | 13.8 | 10.5 | 12.0 | 7.1 | 8.0 | 7.1 | 11.5 | 14.4 | 10.5 | 9.3 |
| **Employment status** | |  |  |  |  |  |  |  |  |  |  |  |  |  |  |  |  |  |  |
|  | Managerial | 29.6 | 28.1 | 17.3 | 14.3 | 57.6 | 38.5 | 30.6 | 34.6 | 39.3 | 40.4 | 39.5 | 22.7 | 38.5 | 33.7 | 20.0 | 28.8 | 46.6 | 38.9 |
|  | Intermediate | 12.7 | 8.3 | 6.1 | 4.5 | 7.3 | 10.4 | 152 | 5.8 | 12.2 | 14.9 | 14.8 | 5.8 | 8.0 | 6.3 | 6.7 | 9.9 | 16.0 | 15.0 |
|  | Small Employers | 9.1 | 5.8 | 3.0 | 3.9 | 3.3 | 6.5 | 5.3 | 9.8 | 6.3 | 6.9 | 8.6 | 19.5 | 4.4 | 14.7 | 4.8 | 4.5 | 6.7 | 8.8 |
|  | Routine/ Semi-routine | 29.4 | 42.2 | 61.8 | 63.5 | 20.4 | 16.3 | 32.4 | 24.2 | 23.6 | 19.6 | 23.5 | 26.2 | 32.1 | 21.0 | 56.3 | 39.3 | 16.2 | 22.4 |
|  | Never Worked/ Unemployed | 9.1 | 7.7 | 7.0 | 8.1 | 6.3 | 9.2 | 7.6 | 11.0 | 9.1 | 6.9 | 5.9 | 10.6 | 7.0 | 14.2 | 7.1 | 8.4 | 5.0 | 5.4 |
|  | Students | 9.9 | 8.0 | 4.9 | 5.8 | 5.1 | 18.2 | 8.9 | 14.6 | 9.6 | 11.3 | 7.6 | 15.2 | 10.0 | 10.2 | 5.2 | 9.1 | 9.5 | 9.5 |
| **Car Availability** | |  |  |  |  |  |  |  |  |  |  |  |  |  |  |  |  |  |  |
|  | 0 | 13.3 | 22.5 | 23.1 | 22.0 | 22.9 | 14.6 | 20.1 | 20.1 | 23.4 | 12.9 | 15.2 | 25.9 | 25.0 | 22.2 | 27.0 | 29.7 | 17.7 | 11.4 |
|  | 1 | 32.2 | 45.9 | 54.7 | 49.5 | 49.5 | 35.7 | 39.8 | 40.4 | 39.6 | 35.5 | 35.7 | 33.8 | 48.9 | 40.9 | 48.6 | 39.5 | 37.8 | 34.8 |
|  | 2 or more | 54.5 | 31.6 | 22.1 | 28.5 | 27.6 | 49.8 | 40.1 | 39.5 | 37.0 | 51.5 | 49.1 | 40.3 | 26.1 | 36.9 | 24.5 | 30.8 | 44.6 | 53.8 |
| **Housing Tenure** | |  |  |  |  |  |  |  |  |  |  |  |  |  |  |  |  |  |  |
|  | Owns Outright | 75.0 | 35.7 | 12.8 | 18.9 | 44.2 | 65.0 | 57.0 | 54.5 | 47.0 | 55.2 | 66.5 | 57.8 | 39.7 | 54.5 | 20.5 | 39.7 | 58.25 | 69.4 |
|  | Private Renting | 12.1 | 54.3 | 77.7 | 71.1 | 52.3 | 27.3 | 26.3 | 30.7 | 36.8 | 33.8 | 25.7 | 27.0 | 51.3 | 35.4 | 71.5 | 48.4 | 32.36 | 22.1 |
|  | Social Renting | 12.9 | 10.1 | 9.5 | 10.0 | 3.5 | 7.7 | 16.7 | 14.9 | 16.2 | 11.0 | 7.8 | 15.2 | 9.0 | 10.2 | 8.0 | 12.0 | 9.38 | 8.5 |
| **Urban – Rural Living** | |  |  |  |  |  |  |  |  |  |  |  |  |  |  |  |  |  |  |
|  | Urban | 19.8 | 25.2 | 22.8 | 4.7 | 46.0 | 29.9 | 21.1 | 40.4 | 35.0 | 23.2 | 25.6 | 39.7 | 32.2 | 36.4 | 19.1 | 20.4 | 24.0 | 22.9 |
|  | Intermediate | 46.6 | 59.3 | 64.1 | 76.5 | 47.9 | 39.8 | 59.3 | 47.4 | 48.3 | 55.9 | 50.3 | 52.4 | 56.4 | 50.6 | 64.9 | 65.8 | 54.8 | 50.3 |
|  | Rural | 35.6 | 15.5 | 13.1 | 18.8 | 6.2 | 30.3 | 19.6 | 12.2 | 16.7 | 20.9 | 24.1 | 7.9 | 11.4 | 13.1 | 16.1 | 13.8 | 17.2 | 28.8 |
|  |  |  |  |  |  |  |  |  |  |  |  |  |  |  |  |  |  |  |  |

Table S3: Prescriptions by migrant group compared to the settled majority

|  | | All prescriptions^1^  *n*(%) | Anti-depressants  *n*(%) | Anxiolytics and hypnotics  *n*(%) | Anti-psychotics  *n*(%) |
| --- | --- | --- | --- | --- | --- |
| **Settled Majority** (total *n*=970,417) | | 233,266 (24.04) | 183, 675 (18.93) | 119,355 (12.30) | 23,905 (2.46) |
|  | **All Migrants** (total *n*=49,342) | 5,608 (11.69) | 4,064 (8.47) | 2,723 (5.68) | 450 (0.94) |
|  | Poland | 1,020 (8.33) | 707 (5.77) | 484 (3.95) | 56 (0.46) |
|  | Lithuania | 335 (7.36) | 237 (5.21) | 151 (3.32) | 23 (0.51) |
|  | India | 180 (6.19) | 109 (3.75) | 101 (3.47) | 15 (0.52) |
|  | USA | 414 (19.12) | 321 (14.83) | 170 (7.85) | 40 (1.85) |
|  | Germany | 676 (25.34) | 529 (19.83) | 353 (13.23) | 67 (2.51) |
|  | North Africa and Middle East | 169 (14.47) | 123 (10.53) | 77 (6.59) | 13 (1.11) |
|  | C/E/W Africa | 304 (17.49) | 216 (12.43) | 153 (8.80) | 32 (1.84) |
|  | Southern Africa | 237 (19.64) | 171 (14.17) | 113 (9.36) | 21 (1.74) |
|  | The Americas/Caribbean | 491 (21.49) | 385 (16.85) | 241 (10.55) | 36 (1.58) |
|  | China and Hong Kong | 190 (7.24) | 119 (4.53) | 103 (3.92) | 17 (0.65) |
|  | C/E/SE Asia and E. Europe | 345 (8.13) | 222 (5.23) | 194 (4.57) | 21 (0.49) |
|  | Southern Asia | 149 (15.31) | 99 (10.17) | 70 (7.19) | 19 (1.95) |
|  | C/E Europe (CEE) | 343 (6.67) | 242 (4.71) | 144 (2.80) | 22 (0.43) |
|  | Southern Europe | 490 (18.82) | 386 (14.83) | 236 (9.07) | 41 (1.58) |
|  | Northern and Western Europe | 193 (14.04) | 147 (10.69) | 96 (6.91) | 12 (0.87) |
|  | Oceania | 265 (18.34) | 198 (13.70) | 133 (9.20) | 27 (1.87) |

C Central, E Eastern, SE South Eastern, W Western

^1^*n* with at least one prescription in the index period
